# Beyond clinical skills: student-reported impacts of a veterinary public health externship in rural Alaska

**DOI:** 10.3389/fvets.2025.1613867

**Published:** 2025-12-19

**Authors:** Laurie Meythaler-Mullins, Allyce H. Lobdell, Caroline Kern-Allely, Danielle M. Frey

**Affiliations:** 1 Colorado State University, Fort Collins, CO, United States; 2 College of Veterinary Medicine and Biomedical Sciences, Colorado State University, Fort Collins, CO, United States

**Keywords:** veterinary students, rural medicine, access to care, experiential learning, public health, veterinary medicine, veterinary education, Alaska

## Abstract

Preventive medicine and public health are critical components of veterinary curricula, requiring students to understand their role in the health of communities and people beyond their animal patients. However, students considering a career in public health often face gaps in the depth of this curriculum. From 2019 to 2024, students from Colorado State University (CSU) and its University of Alaska Fairbanks (UAF) partner participated in the Hub Outpost Project (HOP) externship’s community visits to Yukon-Kuskokwim Delta communities in rural Alaska. The curriculum emphasized veterinary professional concepts, surgical and clinical experience, community engagement and cultural awareness, and self- and team- mindfulness. Through repeated practice and direct exposure to providing care, students improved a variety of clinical and professional skills. Student impacts were assessed through a survey of the participating veterinary students from both institutions. Students primarily reported impacts related to gaining clinical and communication skills, the breadth of human-animal bonds, engaging directly with clients and patients, and realizations and understandings that quality medicine is possible with limited resources. These findings suggest that experiential learning not only greatly improves skills for students but also engages students in areas of veterinary medicine that need an increased workforce, such as rural medicine, while increasing their understanding of diverse community needs.

## 1 Introduction and background

Preventive medicine and public health are important parts of veterinary medicine curricula, evidenced by their inclusion in the American Association of Veterinary Medical College’s (AAVMC) Competency-Based Veterinary Education (CBVE) (1). It is essential for veterinary students to understand the role they can play in community health beyond individual animal care. Despite this critical need, veterinary curricula often focus on the needs of pet owners with regular access to care, leaving students with limited exposure to underserved or resource-limited settings. Students primarily participate in clinical rotations within teaching hospitals, creating an experiential gap for those interested in public health. The best practice to address this gap is to encourage students to partake in as many external experiences as possible, including electives, in which they can also receive mentorship (2). This use of experiential learning promotes an integrated learning approach in veterinary education (3).

Outreach medicine creates valuable experiential learning opportunities that increase preparedness and complement didactic, laboratory, and case-based teaching (4, 5). These opportunities require ethical engagement with communities, as outlined by the *2024 Principles of Veterinary Community Engagement* (5). The first three principles, briefly paraphrased as becoming knowledgeable about the community, building relationships and trust, and creating community partnerships, are particularly relevant. Communities across the US are diverse with diverse needs; for instance, a rural, remote Tribal community with a history of oppression and exploitation faces challenges distinct from a suburban, predominantly white affluent community. While debate exists about a national veterinary shortage (6, 7), rural areas face well-recognized deficits in access to veterinary care (8). With more than one out of four families struggling to access veterinary care across the United States (9), there are additional nuances to barriers in rural areas, including as geographic obstacles and fewer resources (10).

Effective community engagement projects involving outsiders require relationships and trust be built prior to student involvement (11). Partnerships take time, demonstrated reliability, transparency, and thoughtful communication. It is critical to be open to hearing, understanding, and absorbing unique details of individuals and communities. When community-identified needs are prioritized, the project can support “experiential learning in animal healthcare *and* respectful interaction with diverse people” (5, p. 8). Exposure to underserved areas may change students’ perceptions of career choices, potentially encouraging them to consider this type of work and impacting workforce shortages (12). Addressing access to veterinary care in rural regions is essential not only for animal health, but also to protect public health. Preventive veterinary care aligns with the One Health concept, which recognizes the interconnected nature of human, animal, and environmental health as well as the need for cross-disciplinary collaboration (13). Preventive medicine can reduce human health risks posed by human-animal contact, as well as protect human-animal bonds. Lack of veterinary care thus negatively influences public, human, and animal health in multiple ways (3).

To address lack of access to veterinary care in rural Alaska (14) and expand student exposure to rural, public health practice, Colorado State University (CSU) and University of Alaska Fairbanks (UAF) designed and implemented the Hub Outpost Project (HOP). Previous publications from the HOP team have discussed program impacts, including community perspectives and program integration into local health frameworks (10, 11). Our objective here is to explore student’s self-reported motivations, learning experiences, and long-term impacts from participating in HOP externships.

### 1.1 The Yukon-Kuskokwim Delta

**Figure 1:**
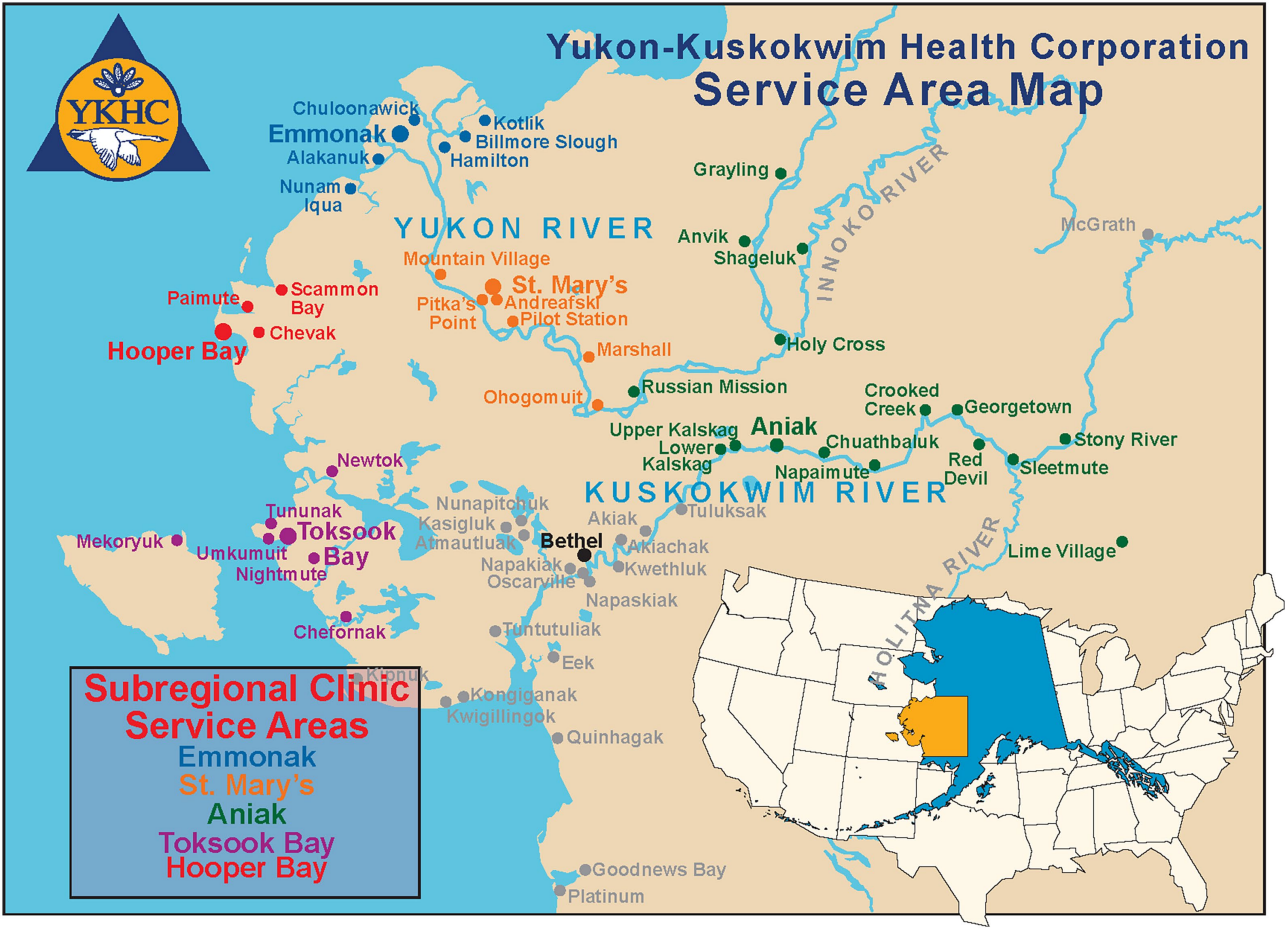
Yukon Kuskokwim health corporation service map.

The Yukon-Kuskokwim (YK) Delta region of southwest Alaska (Figure 1) is remote, rural, and sparsely populated primarily by federally recognized Tribes (10, 15, 16). Residents of this region experience dramatic health disparities compared to other communities on Alaska’s road system. Life expectancy is 10 years less than the US average, driven by cancer, chronic diseases, high rates of infectious disease, and high levels of injuries, including dog bites (17). Human healthcare is provided by the Yukon Kuskokwim Healthcare Corporation (YKHC) which works with Indian Health Services (IHS).

These organizations investigate many dog bites annually to prevent rabies transmission (17). Rabies is the most pressing zoonotic illness in the region which impacts humans as their dogs may encounter the rabies enzootic foxes. Due to the high prevalence of rabies and large number of free-roaming pets, dog bites pose a greater risk in rural Alaska than in other areas in the US. Dogs are prevalent in this region (10) with little access to veterinary care (11, 14), and when access to veterinary medicine is not readily available, the chances of adverse health consequences increase, with widespread impacts on physical, emotional, and financial wellbeing (11).

The American Veterinary Medical Association (AVMA) recognizes the significance of the human-animal bond across societies (18). In the YK Delta, dogs have a distinct and special role in Alaska Native culture, serving not only as companions but also in assisting with transportation, protection, and hunting (14). As Alaskan communities evolve, dogs remain in communities for teaching “Native ways of knowing,” referring to the “deep, holistic, and generational knowledge systems developed by Indigenous Peoples through their intimate relationship with their environment and community” (19). Dog mushing, a tradition in Alaskan culture, is an example of the special role dogs hold in this area. Today, the dog mushing community in the YK Delta has multiple established races, including the Kuskokwim 300, the world’s premier middle distance sled dog race (20). Further, dogs have been used to connect indigenous youth to their heritage, in suicide prevention, and substance abuse programs (21).

### 1.2 The Hub Outpost Project (HOP)

To understand the context of student impacts reported, an understanding of the HOP program and student learning goals are important. The HOP aims to address the complex issue of immediate and future veterinary needs in the YK Delta using a One Health framework (22). We developed and implemented HOP through a founding collaboration between veterinarians Dr. Danielle Frey at CSU, an author on this paper, and Dr. Arleigh Reynolds at UAF. Using a hub-and-spoke design based on the YKHC’s human health model, the project deploys a veterinary team from the “hub” of Bethel to strategically identified “spoke” communities. Each “spoke” community is further accessible to an additional five to ten communities.

Aware of historical exploitation of Tribal communities by outsiders, four initial trips to the region took place to build relationships, trust, and a deeper knowledge of the communities and their needs related to veterinary care. Project objectives were collaboratively developed with key community members, including improving community health through preventive medicine, engaging clients and communities, and veterinary student learning (Supplementary Appendix 1). We launched clinical operations in 2019 with the hire of a full-time Community Outreach & Public Health Veterinarian (COPHV), Dr. Laurie Meythaler-Mullins, an author on this paper. Dr. Meythaler-Mullins built relationships and trust with the community while living first in Bethel and subsequently in Fairbanks and working alongside community health aides and village safety officers who provided real-time communication and feedback.

#### 1.2.1 HOP educational program and the student experience

The externship’s primary goals were to engage students with relevant career interests, encourage practice in underserved areas, and to teach rural medicine and community engagement skills. Student learning objectives included veterinary professional concepts, surgical and clinical experience, community engagement and cultural awareness, and self- and team-mindfulness (Supplementary Appendix 1). Students engaged directly with communities lacking accessible care for their animals while developing skills in mindful community interactions. Students were also exposed to rural medicine, potentially encouraging them to consider this type of work (12). Future veterinarians learned to deliver quality care in under-resourced communities while reflecting on access to care challenges and potential solutions they can incorporate into their own careers.

Each externship typically consisted of two to five students and lasted approximately ten days. Each rotation included three clinic sites, Bethel and two villages, with approximately three days at each. Teams traveled from Bethel to villages by either small aircraft, or, during summer, by boat via the Kuskokwim River. Clinic gear, supplies, and food were weight restricted due to transportation. Accommodations were modest and occasionally required sleeping on the clinic floor. The veterinary clinic was set up in a central communal space, such as a bingo hall or community center. The team adapted these spaces for patient intake, surgery, and vaccine administration to offer walk-in, preventive medicine services. Weather permitting, teams conducted door-to-door visits through the village offering vaccination services.

On average, clinics served approximately fifty animals. Each clinic averaged twenty spay/neuter surgeries. CSU fourth-year students fully participated in surgeries as appropriate to their skill level, confidence, and goals. UAF first- and second-year students’ participation in surgery was restricted, as they had not completed surgical skills education due to their chronological position in the veterinary curriculum. Additional services included rabies and combination vaccines (DA2PP/FVRCP), and broad-spectrum antiparasitic treatments (milbemycin oxime + praziquantel). Clinics also treated common conditions such as skin, ear and eye infections, lacerations, and provided humane euthanasia services. All students participated in client communication and patient care. Students were responsible for obtaining patient histories, explaining treatment plans, conducting patient exams, surgery prep, monitoring and recovery from surgery, vaccine administration, and discharging patients.

#### 1.2.2 Tudent selection and preparation

From 2019 to 2024, the Hub Outpost Project offered a veterinary student externship for participation in community clinics. Student participation differed based on enrollment in either CSU-UAF “2 + 2” veterinary program or the full-time CSU veterinary program. UAF students were offered HOP externships during their pre-clinical years, while CSU students were selected through an Off-Site Learning Opportunity application process for their clinical year. We carefully selected and trained student participants to mitigate any harm to the communities in partnership with the HOP. Due to its small cohort size, the entire UAF cohort was offered a chance to participate. The UAF cohort had a large portion of Alaskan residents who were familiar with the challenging realities of rural life and community compositions in Alaska. Further, the program’s COPHV was able to know each UAF student personally and could intervene if any cause for concern arose, though none did. The CSU cohort’s application process intentionally screened for signs of saviorism and assessed alignment with prospective careers.

All students received information on workloads, logistics, physical activity, clinical, daily and professional life (Supplementary Appendix 2; Table 1). They were instructed on culturally aware engagement through three main mechanisms. First, students were screened for perspectives such as saviorism or other potentially harmful themes. Second, students received pre-departure materials, including YK Delta geography, demographics, and HOP background. Embedded within the materials were cultural components emphasizing respect, judgment-free care, and awareness of barriers to care. Included materials were created in collaboration with local community members to include concepts sensitive to and inclusive of the Yu’pik language and local spiritual and cultural beliefs. These materials specifically state: “The state of human and animal lives with whom we work are very different from those you may have previously experienced. I do demand my externs keep an open mind and heart during your time here, and responsibly and respectfully represent veterinary medicine.” Third, students received background on individuals, communities, and the historical context of animals through structured discussions and informal conversations with local community members. Leaders modeled respectful norms, such as removing gloves for handshakes, sunglasses when meeting people, and how to enter arctic entryways and homes appropriately. Daily debriefs, along with guidance and mentorship, allowed for self-reflection and review of activities.

**Table 1:** Summary of information provided to veterinary students on HOP physical activity, clinical, daily and professional life.

Category	Provided information
Physical activity and clinical life	Carry dogs awake and anesthetized. Dogs may weigh upwards 40 kg, you will not be expected to carry them alone, but to make a smart plan in transportation
	Experience extremes in temperatures, primarily cold
	Interact with, restrain dogs and cats who might be afraid, nervous, and reactive. Be prepared to prevent injury to yourself and your team
	Stand and perform multiple surgeries in a day
	Stand for extended period of time, possibly on hard surfaces
	Walk on uneven ground
Daily personal and professional life	Be prepared to patiently wait and readjust schedules
	Reside in housing with various levels of shared spaces
	Stretch skills in adaptation
	Travel closely with a small group of people where you will likely have to ask for help, share emotions, and communicate about themselves in a group setting
	Work in various locations with variable control of the environment, the temperature and air movement, and distance to bathroom

## 2 Materials and methods

### 2.1 Survey development and sampling

We developed a survey to gather information regarding the experiences and impacts of HOP on its participants using the online survey platform Qualtrics. The survey was comprised of five sections: (1) screening questions to verify HOP externship participation, (2) motivations for participation, (3) experiential feedback, (4) long-term impacts from participation, and (5) demographic and background information. Many questions included both multiple-choice and open response options (Supplementary Appendix 3). We consulted with CSU Director of Human Research Protection Program and reviewed the Institutional Review Board (IRB) Human Research Determination worksheet to assess if formal IRB approval was required. This evaluation did not meet the criteria for human subjects requiring IRB review. All participants were provided fully informed consent statements.

In March 2024, we piloted the survey with two past HOP participants. In April 2024, we invited all previous HOP student participants to take the finalized survey. Externships continued through August of 2024, with those additional participants invited to take the survey once their rotation concluded. In total, all but two students with missing contact information were invited to take the survey (*n* = 99). The survey was closed in September 2024.

### 2.2 Analysis

Survey data was exported from Qualtrics into Microsoft Excel format and de-identified for analysis. Quantitative analysis, consisting of descriptive statistics for the multiple-choice responses, was performed in RStudio version 9.1.401. Qualitative analysis, for the open responses, was performed in Excel. A thematic analysis approach was used for open-text response questions. This process was iterative and involved three cycles of coding to capture concise and accurate themes. Prior to coding, all responses for each question were read to establish clear understanding of each response. The first coding cycle used a detailed “splitting” approach to capture as many details as possible (23). The second coding cycle included reviewing codes to identify conceptually similar codes and consolidate them into smaller, coherent sets of codes. The final coding cycle grouped codes to produce the final high-level themes. These themes were reviewed by two independent coders for consistency. Frequency of themes were calculated.

## 3 Results

### 3.1 Respondent demographics

Of the 99 students contacted, 49 completed at least part of the survey (54% response rate) and 40 completed it in full (40% completion rate). Eighteen respondents indicated enrollment in the UAF “2 + 2” program and 22 indicated enrollment in the CSU 4-year DVM program. Demographic characteristics of respondents are presented in Table 2. The average participation age was 28 (range 22–36). Respondents primarily identified as White, with smaller proportions identifying as Latino, Black, Asian, or other races/ethnicities. Most respondents identified as women (93%).

**Table 2:** Survey participants’ self-reported demographics.

Participant age (n = 38)	
Mean (SD)	27.92 (3.49)
Median (Range)	28 (22, 36)
Number of respondents by year of participation (n = 49)	
2019	1
2020	2
2021	1
2022	12
2023	16
2024	17
Gender, n (%)	
n	40
Woman	37 (92.5%)
Man	3 (7.5%)
Race/ethnicity, n (%)	
*n*	43
White	37 (86%)
Black	6 (14%)
Latino/a/e	1 (2%)
Asian	1 (2%)
Other groups	1 (2%)
*Race/ethnicity was a multiple-select response option. The total of all categories will not equal N.	
Socioeconomic status, n (%)	
*n*	40
SES – Upper class	1 (2.5%)
SES – Middle class	28 (70%)
SES – Working class	11 (27.5%)
SES – Lower class	0 (0%)

### 3.2 Clinical veterinary skills gained

All respondents (*n* = 49) reported gaining at least one clinical skill, with most reporting 3 to 6 skills. The most frequently reported skill was teamwork (*n* = 47, 96%), followed by surgical skills (*n* = 42, 86%), client communication (*n* = 38, 78%), patient handling (*n* = 32, 65%), collegial communication (*n* = 28, 57%), and vaccine administration (*n* = 24, 49%). Other skills (*n* = 8, 16%) included physical exam skills, flexibility, patience, stress management, discharge skills, and confidence. Topics surveyed, qualitative themes identified, and an illustrative quote for each topic are presented in Table 3.

**Table 3:** Student-reported (*n* = 49) impacts on veterinary clinical skills from participation in the HOP externship.

Veterinary clinical skills	Students *n* (%)	Top themes (*n*)	Select quotes (themes)
Teamwork	47 (96%)	**Skills used and/or improved**	*“It was a small group of us (2 students and 1 vet) that were able to manage not only the medical/anesthesia/surgical part of the trip but also the logistics/packing/sterilizing parts. Additionally, it showed how critical local partners were for success with trips like this.”* (Coordination of tasks)
		Supporting the team (10)	
		Communication (5)	
		Problem solving (5)	
		Coordination of caseload (3)	
		**Teamwork drivers**	
		Coordination of tasks (12)	
		Clinic efficiency (10)	
		High volume (5)	
		Limited conditions (5)	
		Team size (5)	
Surgical skills	42 (86%)	Practice opportunity (23)	*“This rotation was HUGE for my surgical skills. Both 4th year veterinary students performed 2–4 surgeries per day, the majority being OVHs. Both Dr. Boyd and Dr. Laurie created a calm and supportive environment. The environment further helped me feel comfortable and confident even when things went awry.”* (Condensed practice opportunity; Instruction; Confidence)
		Confidence (12)	
		Instruction (11)	
		Learn techniques (7)	
		Clinic site (5)	
		Condensed practice opportunity (4)	
Client communication	38 (78%)	Direct client interaction (13)	*“There is a broad range of client knowledge out in the villages, some people are very well informed about what we do, and for others this is the first time they have ever brough[t] their pets to a vet. So having to gauge each persons understanding and adjusting to each different person can be difficult but VERY rewarding, and definitely tests your client communication skills.”* (Exposure to diverse set of clients; Diverse client needs; Client education)
		Exposure to diverse set of clients (9)	
		Diverse client needs (8)	
		Client education (7)	
		Empathy/respect for clients and communities (6)	
Patient handling	32 (65%)	Engaged with a variety of temperaments (13)	*“[The externship] definitely improved [my patient handling skills]. As someone that had predominantly worked in specialty medicine it showed me a different side of how we can also safely handle patients in a higher volume. It also showed me that even without advanced monitoring supplies, patients can do quite well for heavily sedated procedures.”* (Worked with limited tools)
		Had lots of opportunity to engage (9)	
		Engaged with diverse patients (6)	
		Engaged with unsocialized dogs (5)	
		Completed physical exams (4)	
		Worked with limited tools (4)	
Collegiate communication	28 (57%)	Teamwork necessitated communication (19)	“*This experience enhanced collegial communication though the fast paced environment to ensure patients were taken care of properly and through overnight stays with colleagues which emphasized respecting others needs”* (Teamwork; Environment)
		Environment impacts (e.g., chaotic, stressful, busy) (5)	
		Peer learning (4)	
Vaccine administration	24 (49%)	Engaging in high volume work (10)	“*When doing door-to-door vaccines, we were sometimes tasked with vaccinating aggressive or fearful dogs that could not be handled in the typical way I had been trained. This required careful timing and planning, as well as efficient and confident use of the needle and syringe to ensure the safety of the crew and our patient, without escalating the situation”* (variety of situations)
		Increased efficiency of vaccination skills (6)	
		HOP provided the ability to practice (3)	
		Engaged in a variety of situations (2)	
Other	8 (16%)	Conducting physical exams (3)	“*Managing stress in clinic, going with the flow, being grateful to be a veterinarian, and being a better person overall. Very few people get to see this part of the world from a veterinary perspective and from a human perspective. It has changed my outlook for the better on being a veterinary practitioner and admittedly made me a better person.”* (flexibility)
		Having flexibility (2)	

Forty-seven (89%) respondents expanded on teamwork skills. Twenty-seven distinct themes emerged, falling into two main categories: (1) teamwork skills used or improved (e.g., communication, problem-solving, team support) and (2) drivers of teamwork (e.g., maintaining clinic efficiency, coordinating tasks and high caseloads, working in limited conditions). For surgical skills, the primary theme identified by respondents was the opportunity to gain surgical experience within a variety of clinic sites and environments, with several participants noting condensed time and high case load. Another major theme under surgical skills was “instruction,” referencing the COPHV’s close guidance and supervision, encouragement to step outside of comfort zones, and creation of a safe space to practice skills. Respondents who reported not gaining surgical skills were overwhelmingly UAF students, consistent with their earlier position in the DVM curriculum.

Forty (82%) respondents expanded on client communication skills. Themes highlighted direct client interactions, exposure to diverse clients, ability to address a variety of client needs, client education, and empathy and respect for clients and communities. Twenty distinct themes emerged for patient handling skills. The primary theme noted was exposure to diverse patients with a variety of temperaments, as well as the frequent hands-on opportunities, allowing for different types of handling. Notably, several respondents indicated that working with limited resources was an important aspect of gaining patient handling skills. Thirty-six (75%) respondents expanded on collegial communication skills, emphasizing close team proximity as critical for communication. Twenty-one (44%) respondents highlighted high volume as the primary theme for vaccine administration skills, as well as diversity of patients.

### 3.3 Tools gained to work in rural spaces

All respondents (*n* = 40) reported gaining at least one practical tool for working specifically in rural spaces (Table 4). The most frequently reported was the ability to practice in resource-limited situations. Open ended response options were not provided for this section.

**Table 4:** Student-reported (*n* = 40) tools for rural spaces gained from participation in the HOP externship.

Tools to work in rural regions	Number of students reporting *n* (%)
Ability to practice in low-resourced situations	34 (85%)
Ability to work in nontraditional clinic spaces	32 (80%)
Ability to work in geographically isolated spaces	29 (73%)
Client connection and rapport	28 (70%)
Confidence	27 (68%)
Surgical Skills	27 (68%)
Communication	23 (58%)
Professional collaboration	22 (55%)

### 3.4 Impacted perceptions and knowledge bases

All respondents (*n* = 49) reported their perception or knowledge base of a variety of topics was impacted through their participation in the externship (Table 5). Nearly all respondents (*n* = 45, 98%) reported that their perceptions or knowledge of working with limited resources was impacted. Common qualitative themes in this category included versatility of minimal resources, learning adaptability, and the capability of providing high-quality care with resource limitations. Eighty-seven percent (*n* = 40) of respondents reported an impact on working with rural communities. Common themes here included community gratitude, community enthusiasm to engage with the HOP team, and respect for diversity. Additionally, respondents referenced mistrust of veterinarians and noted witnessing barriers to care despite need. Eighty percent (*n* = 37) also reported an impact to their understanding of resource-restricted, geographically isolated, and nontraditional clinical spaces. Students noted surprise at the ability to improvise clinics and to practice quality medicine in unconventional settings.

**Table 5:** Student-reported (*n* = 49) impacts to perceptions or knowledge of topics from participation in the HOP externship.

Topics	Students *n* (%)	Top themes (*n*)	Select thematic response
Working with limited resources	45 (98%)	Versatility of (14)	*“I am now an advocate for working with limited resources after this externship. It required more application of clinical skills and critical thinking. We were also able to provide excellent quality of care to these dogs. Even with our protocol and discharging them shortly after their procedures, they were walking out of the building and comfortable.”* (quality medicine with)
		Ability to adapt (11)	
		Quality medicine with (10)	
		Ability to provide care (8)	
Working in rural communities	40 (87%)	Knowledge of/respect for diverse communities (12)	*“It was eye opening to see how people live in the rural villages we visited. I am familiar with how Indigenous Native Americans live and are treated in New Mexico, having grown up in an area surrounded by pueblos and reservations, so this was another iteration of that but in it’s own - more extreme - Alaskan style; subsistence lifestyle as a necessity. It was humbling to learn of the history of ongoing colonialism in the region, and see how the effects of colonialism, racism and capitalism are still present and ongoing. Despite this, we were warmly welcomed by the locals. Participating in this trip helped cement the idea that while we cannot change the past, we can still act to heal from it and create a better future, and I can do it as a vet (which is cool)!”* (Knowledge of/respect for diverse communities; Specific community characteristics)
		Specific community characteristics (12)	
		Exposure to/knowledge of AVC (8)	
		Differing human-animal bonds (5)	
		Exposure to/respect for rural medicine (7)	
		General exposure to rural communities (4)	
Resource restricted, geographically isolated, and nontraditional clinic spaces	37 (80%)	Improvised clinic spaces (6)	*“I had never imagined practicing medicine from a bingo hall, or sleeping in a traveling medical clinic while providing care. It was awesome to see the community willingly help us find spaces to provide care, and taught me that creative thinking can make the most unusual places effective medical care facilities if done properly”* (improvised clinic spaces)
		Ability to still practice quality medicine (5)	
Public health	30 (65%)	Human-animal connections (16)	*“The public health risk of rabies exposure to dogs in the communities, and therefore to people, left a huge impact on me, because in the non-rural areas of the lower 48 that I’m used to, rabies exposure is so incredibly rare this is not something we think about day to day. It was eye opening to serve a community where something that feels as simply as a rabies vaccine is actually making a huge impact on public health and transmission of zoonotic disease. It also opened my eyes in general to how interconnected human and animal health is, and how essential veterinarians are in the “one health” world of medicine and in serving rural communities alongside physicians, dentists, etc.”* (human-animal connections; presence of rabies; vet med/public health connectedness)
		Presence of rabies (7)	
		Vet med/public health connectedness (5)	
Zoonotic diseases	20 (43%)	Rabies (in general) (17)	*“We learned about the importance of rabies vaccinations due to the high prevalence in the fox population. Since veterinary access is limited, we also learned about administering rabies yearly/at every clinic that is provided to assure animals and humans are both protected. The other zoonotic disease we discussed was echinococcus which can cause hydatid cysts in humans. This parasite is targeted by praziquantel which is not always included in the more common dewormers. This emphasized the importance of knowing which zoonotic diseases exist in where you practice medicine and how to treat them.”* (Prevalence of rabies in AK; Importance of preventive med)
		Prevalence of rabies in AK (9)	
		Importance of preventive medicine (vaccinations) (6)	
		Public health threats (5)	
Food insecurity	13 (28%)	Food cost/availability (5)	*“This was another eye opening lesson. To walk into the grocery stores and see the lack of* var*iety and the high cost, in addition to seeing people smoking fish they’d caught exemplified the logic and necessity behind the subsistence lifestyle that people there practice. We live hugely privileged lives, especially as majority white, upper-middle class veterinary students that make up the CSU student body, so to be confronted with a way of life that exists so different to our own hopefully inspires those of us who participate to take action (that our privilege allows and demands) to benefit people less privileged than ourselves.”* (Food cost/availability; Subsistence living)
		Distribution of resources in the community (2)	
		Subsistence living (2)	
Epidemiology	7 (14%)	Factors influencing disease (1)	*“Going to a small village you can understand the importance of the distribution, patterns, and causes of disease and health conditions in a population. For these communities, an outbreak can completely decimate the population. A few of the community members shared stories of experiences they had with their pets that resulted in a loss or illness.”* (Severe impact on small communities)
		Rabies epidemiology (1)	
		Rural disease (1)	
		Severe impact on small communities (1)	
		Targeting interventions (1)	
Food Safety	7 (14%)	Risks (1)	*“The locals were extremely kind and would bring us food like dried meats from their hunting season. One of the locals really wanted to share with me dried salmon which was delicious but could not help but think of the meat products that they consume that probably does not have quality control and disease monitoring on prior to consuming. Which their food is also coming from the wild and not a production animal, which has different risks. I was wondering how they learn what meat is safe or not to consume. How do they inspect the carcusses [sic] or do they? Do they have testing that they do?”* (Risks)

Sixty-five percent of students (*n* = 30) reported that participation impacted their knowledge or perception of public health, particularly connections between human and animal health. Qualitative themes on this topic included the public’s engagement with veterinary medicine and the impacts on livelihood of humans via the health of animals. Forty-three percent (*n* = 20) reported on zoonotic disease, with nearly all referencing the prevalence of rabies (Table 5). Many participants mentioned shock over the real public health threat that rabies poses in Alaska, as rabies is generally under control in the contiguous US. A recurring theme was the significant impact of vaccinations.

While few students reported the topics of food insecurity (*n* = 13, 28%) and epidemiology (*n* = 7, 15%) as covered or immediately apparent, those who did provided expanded insights. For food insecurity, major themes included cost and availability of food, as well as how communities adapt through shared resources and subsistence living. Impacts on food safety were reported by 13% of students (*n* = 7), primarily on lack of discussion within the program.

### 3.5 Long-term impacts

All students who answered the question on whether the HOP externship had a lasting impact indicated that it did (*n* = 41). Ninety-five percent (*n* = 39) expanded on those impacts (Table 6) including a desire to continue engaging in similar work (*n* = 21, 54%), impact to confidence/skills (*n* = 16, 41%), career paths (*n* = 13, 33%), and societal and community impacts of the HOP (*n* = 3, 8%). Additional themes included the impact of the mentorship (*n* = 3, 8%), knowledge and application of spectrum of, contextualized, and accessible care (*n* = 3, 8%), human-animal bonds (*n* = 3), and the importance of empathy for all clients (*n* = 2, 5%). Several students indicated how memorable their experience was and that they look back on it with fondness.

**Table 6:** Student-reported (*n* = 39) lasting impacts of participation in the HOP externship.

**Theme** Subtheme	**Number of mentions**
**Career path**	**11**
Accessible veterinary care	6
Rural communities	3
Community/shelter medicine	1
General knowledge	1
Non-traditional jobs in vet med	1
**Continue engaging in similar work**	**9**
Specific region/community	3
Outreach	2
Accessible veterinary care	1
One Health	1
Spay and neuter	1
Unspecified	1
**Confidence/skills**	**7**
Confidence as a veterinarian	3
Clinical skills	2
Confidence to practice with limited resources	2
Confidence in surgery	2
Application of skills gained	1
Interpersonal skills	1
**Impact**	**5**
of community’s gratefulness	3
on society (of clinics/service)	3
**Other Themes**	**23**
Think/talk about often	5
Memorable	4
Changed perspective on vet med	3
Human-animal bonds	3
Mentorship	3
Spectrum of/contextualized care (knowledge of, application of)	3
Having empathy for all clients	2

**Figure 2:**
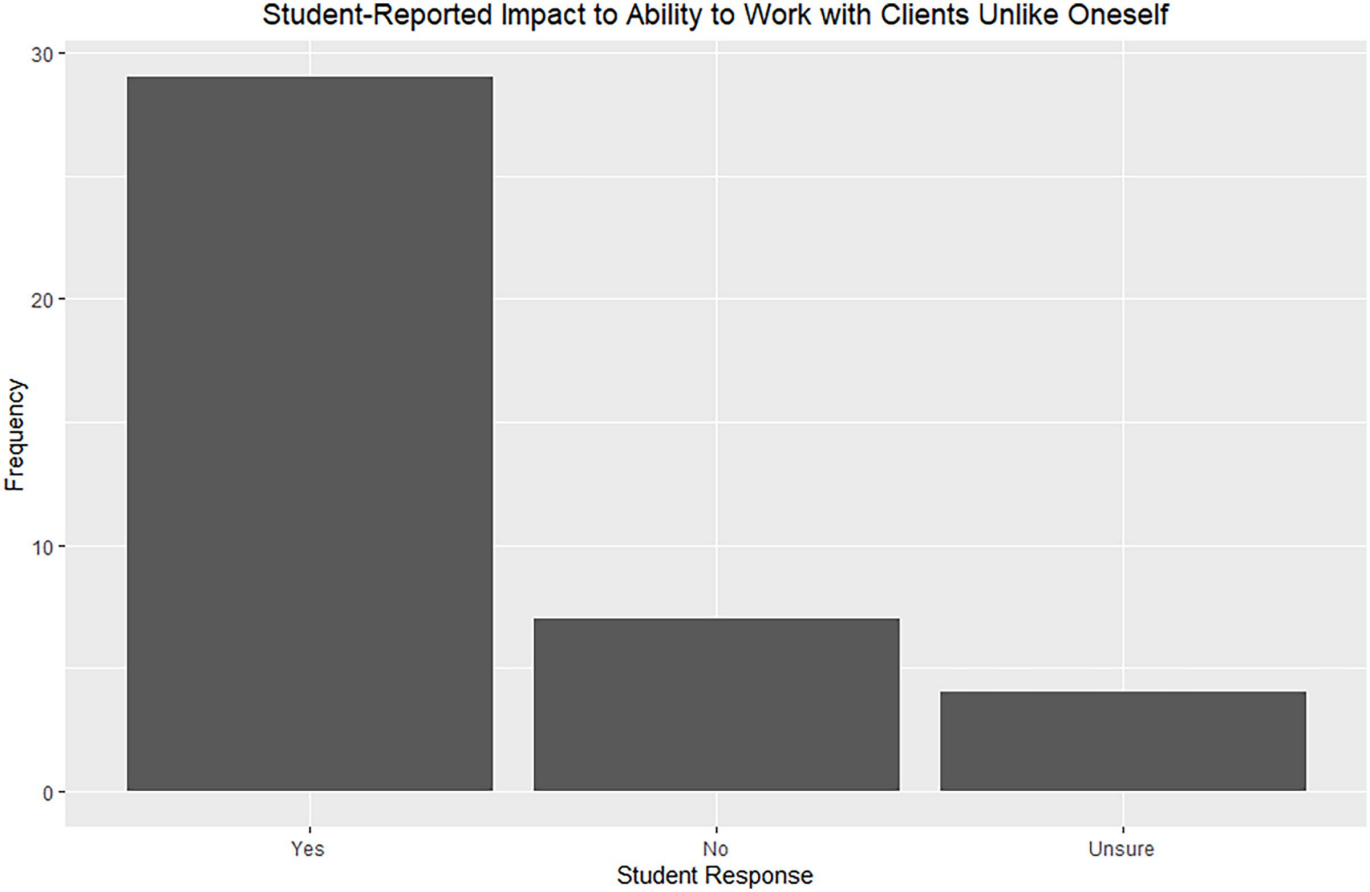
Student-reported impact to ability to work with clients unlike oneself.

When asked whether the externship improved their ability to work with clients from different cultural or socioeconomic backgrounds, most respondents (*n* = 29, 73%) said yes (Figure 2). Thematic analysis (*n* = 34) identified three key outcomes: improved communication tools (*n* = 28, 82%), broader appreciation of human-animal bonds (*n* = 8, 24%), and increased understanding of the need and ability to practice spectrum of care (*n* = 7, 21%). Respondents emphasized how evident it was that clients had just as much love and care for the animals as anyone else, despite bonds and relationships appearing different than in other settings.

Lastly, 31 of 40 respondents (78%) reported that they apply One Health concepts in their current veterinary practice. Common themes reported (*n* = 35) were that awareness of One Health improved (*n* = 21, 60%) and that their understanding of how animal health impacts human health improved (*n* = 15, 43%). Others more general reported that the externship was a good example of One Health in practice (*n* = 14, 40%).

## 4 Discussion

This externship created valuable experiential learning opportunities that increased veterinary student preparedness, complementing programmatic didactic, laboratory, and case-based teaching. HOP externs engaged in an integrated learning approach through repeated practice and direct exposure to real-world contexts. HOP externs had increases in confidence, empathy, and respect for diverse clients, and enhanced understanding of public health.

### 4.1 Learning drivers: repetitive practice and direct exposure

This externship demonstrates that experiential learning is essential to veterinary education and can improve student skill development. When students are immersed in community settings and their unique needs, they experience first-hand why particular skills are essential. Throughout the HOP externship, repetitive practice and direct exposure of skills with multiple patients were key in student learning and mastery building. Students had a focused amount of time, enhanced by a high case load, to engage in and hone hands-on skills.

Surgical, clinical, and communication skills benefited from this approach. Students reported that the ability to receive and implement real-time feedback contributed to building memory and muscle memory for tasks. Students also identified that repeat opportunities were an important factor in gaining surgical skills quickly. For example, HOP externs may perform between five to ten surgeries in as little as three days, whereas a typical CSU DVM student may perform a similar number of surgeries during the entire DVM program. Clinical skills such as patient handling, physical exams, and vaccine administration also improved through repetition. The high volume of the work allowed for skills to be practiced on patients with a variety of temperaments. Communication skills were strengthened through repeated client interactions and team coordination. The HOP externship provides communication experiences that align with recommendations for attaining effective communication skills to successfully transition from university into practice as students interacted with clients, teammates, and community partners of different cultures and backgrounds. Further, the constant proximity of the team during the HOP externship was a critical component for building collegial communication skills. The immersive, high-volume setting necessitated effective communication while mirroring complex dynamics of real-world practice.

### 4.2 Beyond clinical skills: confidence, empathy, and working with limited resources

Learning outcomes of the HOP externship include skills difficult to directly measure, such as confidence, interpersonal skills such as empathy, and the ability to deliver quality care with limited resources. Confidence emerged as a recurring theme in the thematic analysis of student respondents. Students had more confidence in their ability to adapt to resource-limited contexts, their ability to communicate with diverse clients, their ability to gain, share, and apply knowledge. Client communication interactions allowed students to build confidence as they both addressed a variety of client needs as well as provided education on critical topics.

Students also reported deeper empathy and respect for clients and communities unlike their own. Through community engagement, students could also see diverse expressions of the human-animal bond. Students appreciated how people everywhere love their animals, and how there is a shared desire for them to be healthy. Students also noted their significant impact on public and animal health in the community. For example, the threat of rabies was striking for students, yet allowed them to perceive a greater community impact with vaccinations.

Nearly every student reported developing the ability to provide quality care in limited resource settings. This adaptability is critical in addressing veterinary shortages in the rural United States. Through HOP, students learned that effective, compassionate care is possible even when resources are constrained. This aligns with the growing emphasis on accessible veterinary care and spectrum of care frameworks.

### 4.3 Inspiring rural veterinary work

Our program’s educational design aims to expose students to practicing veterinary medicine in rural areas with restricted access to supplies and services, including what it is like to live and work in these regions. Many students wished to continue to serve resource-limited or underserved areas after participating in the externship. While improved technical skills, confidence, and empathy contribute to this interest, the positive instruction and mentorship are equally influential. Students repeatedly emphasized that their learning was enhanced by close, direct, positive instruction, and a safe learning environment that encouraged to work outside of their comfort zones. Working in close quarters with a positive student-instructor ratio, lends to receiving and application of feedback in-time, accessibility to have questions answered, and advice given and received.

### 4.4 Limitations

This paper’s focus is the student-reported impact of the HOP and not the development of the HOP program. Therefore, limitations are discussed in the context of the externship and this study. Externship elements that could be refined for future opportunities include deeper cultural awareness and focused participant selection across cohorts. While cultural awareness was important in program development, the externship curriculum could be strengthened with increased engagement with the history and culture of the Alaska Native tribes served. Additionally, CSU students were screened in the application process while all UAF students were automatically offered a place in the externship, suggesting potential for more robust participant selection for the externship.

Programmatic learning objectives evolved over time as the externship matured and received focused support, such as a USDA grant focused on student experience. Replication of the externship in other contexts may be limited by cost, logistical demands, and unique community partnerships. Yet implementing veterinary externships that serve communities with similar geographic and socioeconomic backgrounds is both feasible and encouraged.

Study limitations include survey timing, self-reported data, the lack of qualitative software for analysis, and potential biases. Some students were surveyed years after participation, others within weeks, potentially introducing bias in recall accuracy as well as appropriateness of long-term impact assessment. Self-reported data are also subject to biases such as selective memory or social desirability. A qualitative software such as NVivo would have been more appropriate for thematic analysis but was not available to the research team at the time of the study. Participation in the externship also contributed selection bias in students interested in the topics addressed. Further, all authors are strong advocates in accessible veterinary care, which may have introduced bias in survey design and subsequent thematic analysis. Finally, the word “impact” was intentionally used throughout the survey as a non-directional neutral term, thus the study did not measure whether impacts were perceived as positive or negative.

### 4.5 Conclusion

Quality veterinary care can and should be delivered in resource restricted regions. Students can be successfully incorporated in partnership with communities. Through these experiential learning opportunities, students can gain and improve clinical, surgical, and communication skills in a short amount of time. This learning is enhanced by repeated practice with an increased case load and direct exposure to diverse patients and clients. Experiential learning can also expose students to areas of the veterinary medicine profession that need an increased workforce, including rural medicine. These experiences produce both immediate and long lasting impacts on students’ professional development.

In the HOP externship, set in rural Alaska providing care to culturally Indigenous and subsistence living communities, student learning went beyond hands-on clinical and surgical skills. Students experienced intercultural client communication in various clinical settings and scenarios. Additionally, team and community engagement skills were enhanced through the inherent nature of the travel and intense living and learning situations.

Students gain notable benefits from this type of learning environment that are good for the future of the profession and care provision in communities that need it. The authors recommend that experiential learning, particularly in underserved areas, continue to be evaluated for outcomes. Future implementations should integrate structured cultural awareness training and continue to improve curriculum development and student learning outcomes.

The authors have provided the official curriculum as delivered (Supplementary Appendix 1) for this externship in hopes that educators and veterinarians can utilize it as a guiding structure, alongside the *2024 Principles of Veterinary Community Engagement* and the CBVE, to partner with communities to build similar educational opportunities. An e-book with specific guidance on building such a program is forthcoming which addresses that all aspects depend on the community being served. Experiential learning rooted in partnership and community engagement benefits students, educators, and the veterinary profession.

## Data Availability

The original contributions presented in the study are included in the article/Supplementary material, further inquiries can be directed to the corresponding author.
