# Supplementary material for: Beyond clinical skills: student-reported impacts of a veterinary public health externship in rural Alaska
Source: Front Vet Sci. 2025 Dec 19;12:1613867. doi: 10.3389/fvets.2025.1613867 (PMC12758408; doi:10.3389/fvets.2025.1613867)
Supplement: Supplementary file 1 [file Data_Sheet_1.pdf]

Experiential Rotation Veterinary Student Curriculum and Approach

Preventative Medicine Campaign Program

Background: The Hub-Outpost Program (HOP) has operated in the Yukon-Kuskokwim Delta region of Alaska since 2019. The HOP tackles the complex issue of access to veterinary care in rural Alaska, through a dynamic approach to addressing immediate and future needs by undertaking actions that range from influencing policy to delivering vaccines. The work of this program engages future veterinarians in situations in which they can learn to deliver quality care in under-resourced communities as well as think about access to care and solutions they can incorporate into their practice or career. The University of Alaska Fairbanks (UAF) /Colorado State University (CSU) Collaborative veterinary program (or "2+2 program") enrolls veterinary students who attend 2 years of school at the Fairbanks campus in Alaska and then complete their veterinary degree at the CSU Fort Collins campus in Colorado. The UAF/CSU Collaborative students had the opportunity to participate in these HOP visits during their time in Alaska and during their fourth year of clinical externships. CSU based veterinary students could participate during their fourth year of clinical externships.

Program Objectives:

- Improve animal and community health through MASH style community clinics, where care is delivered at no cost to pet-owners
- Offer preventive medicine, veterinary services and quality sterilization surgeries for dogs and cats
- Engaging clients and animal owners in communities in the YK Delta in an inclusive, kind and thoughtful manner
- Create an inclusive, safe space for students to learn
- Expose students to the process of providing Preventative Care in communities.
- Expose students to living and working in rural regions with restricted access to supplies and services

Externship structure: Veterinary students accompany a veterinarian on travel to a rural community to provide preventive veterinary care to the animals of the residents in those communities. These communities are off the road system and geographically isolated from access to supplies and health care providers. The team brings all veterinary supplies and equipment needed to deliver vaccinations, parasite control, first line veterinary care and to perform OHE and castration surgical procedures. The team delivers care from a MASH style clinical base in a central community building and goes door to door to deliver vaccinations and parasite control, while registering patients for surgical and other care needs.

Student Learning Objectives: Due to the complex and fully immersive nature of this learning externship, the learning objectives cross multiple categories in professional, clinical and personal themes. Many of these are obtained through overt learning opportunities while some are learned by the inherent nature of traveling, community immersion, and teamwork.

Veterinary Profession Concepts:

- Public health and One Health
- Rabies and zoonotic diseases: transmission and community consequences

- Demonstrate understanding of canine vaccine protocols and antiparasitic treatments
- Exposure to practice veterinary medicine in rural areas
- Understanding of roles of different agencies and departments in safeguarding human and animal health (i.e. USDA, State Veterinarian)

Surgical and Clinical Experiential:

- Experience various surgical approaches to OHE and castration
- Increase confidence in ability to perform OHEs in feline and canine patients
- Increase confidence in ability to perform castrations in feline and canine patients
- Improve efficiency in the processes of preparation, performance, completion and support actions during a day of surgery (e.g. hand movements, body positioning and instrument handling and placement)
- Gain comfort with the anesthetic and medication protocols used in rotation
- Use observational clinical skills and minimal technology to determine patient health, and anesthetic depth and maintenance.
- Calculate and apply peri-operative medications – analgesics and antibiotics.
- Perform intramuscular, subcutaneous and sublingual intravenous injections

Community and Culture:

- Be able to describe variations in how the human animal bond exists and understand that animals may have different roles in their family unit than seen elsewhere
- Engage and communicate with colleagues and members of the community in a manner that is respectful
- Understand and display the principles of being a good guest in a community (greetings, food, comments)
- Prepare to be positive ambassadors of your host community, country and culture upon return

Self and Team:

- Practice self-reflection regarding experienced challenges, growth and progress made
- Engage with teammates in a respectful, thoughtful manner
- Communicate patiently and effectively
- Be able to identify and communicate about preferred communication, feedback and learning methods
- Conscientious use of materials for waste, efficiency and consideration of cost/difficulty to

Topics discussed in small group or "rounds" type settings:

- Community dog and variable concepts of ownership, and demonstration of ownership
    - Free-roaming community owned, free-roaming individually owned, fenced, chained etc.
- Logistics associated with Community Medicine and delivery of MASH style veterinary care
    - Preparation for travel with weight and space restrictions
    - Visiting a community door to door – incorporating community norms and measures of respect

- Partnerships and communication are important to building and maintaining a community-based program
- One Health, Public Health and how animal health and human health are related
- Subsistence living and low-resourced communities, managing food safety and security in a changing climate
    - Botulism. There have been cases in the YK Delta related to fermented fish heads and seal oil
        - **https://yk-health.org/wiki/Botulism**
    - Rural areas and limited access to healthy foods. A common topic brought up by the community and noted in student reflections. National Institutes of Health recognized it as a concern and awarded YKHC a $5 million federal food security grant
        - **https://www.kyuk.org/health/2023-10-02/yukon-kuskokwim-health-corporation-receives-5m-federal-food-security-grant**
    - Climate change- salmon fishing in the Kuskokwim River has been collapsing, and people have not been allowed to fish even for subsistence use. Community members brought this up repeatedly when interacting with students.
        - **https://alaskapublic.org/2023/06/08/salmon-are-disappearing-on-the-yukon-and-kuskokwim-heres-what-to-know-about-the-crisis-this-summer/**
    -

Student Post Travel Project: All participants are asked to submit a one page reflection based on a photo they took during the externship. The students are asked to pick one photo that represents an impactful moment from their time with the community, and reflect on that as well as the other impacts felt during their time.

Student preparation prior to travel: In order to help the team perform, engage community and realize learning objectives, students should be given set expectations and details about what to expect, how to prepare and how the program and travel could go. Students and all team members should be informed, repeatedly, that while every detail of the trip has been organized, scheduled and prepared, the reality is that change, and unpredictability are the norm.

Travel

- Methods of transportation can be commercial airlines, small airplane (2-10 seats), small boats on a river, snow-machines, all-terrain vehicles.

Physical activity and Clinical life:

- Walk on uneven ground.
- Experience extremes in temperatures, primarily cold.
- Carry dogs awake and anesthetized. Dogs may weigh upwards 40 kg, you will not be expected to carry them alone, but to make a smart plan in transportation.
- Interact with, restrain dogs and cats who might be afraid, nervous, and reactive. Be prepared to prevent injury to yourself and your team.
- Stand for extended period of time, possibly on hard surfaces.
- Stand and perform multiple surgeries in a day.

Daily personal/professional life

- Stretch skills in adaptation
    - Work in various locations with variable control of the environment, the temperature and air movement, and distance to bathroom.
    - Be prepared to patiently wait and readjust schedules.
- Travel closely with a small group of people where you will likely have to ask for help, share emotions, and communicate about themselves in a group setting
- Reside in housing with various levels of shared spaces.
