# Supplementary material for: Beyond clinical skills: student-reported impacts of a veterinary public health externship in rural Alaska
Source: Front Vet Sci. 2025 Dec 19;12:1613867. doi: 10.3389/fvets.2025.1613867 (PMC12758408; doi:10.3389/fvets.2025.1613867)
Supplement: Supplementary file 2 [file Data_Sheet_2.pdf]

Dear Veterinary Extern Students,

Here is some information to help prepare you for your upcoming Alaska externship. Please note schedules and timeline in rural Alaska operate different then you may be used to- while we will have an itinerary, one thing that I can guarantee about your rotation- the plan at the beginning not look the same by the end of your rotation!

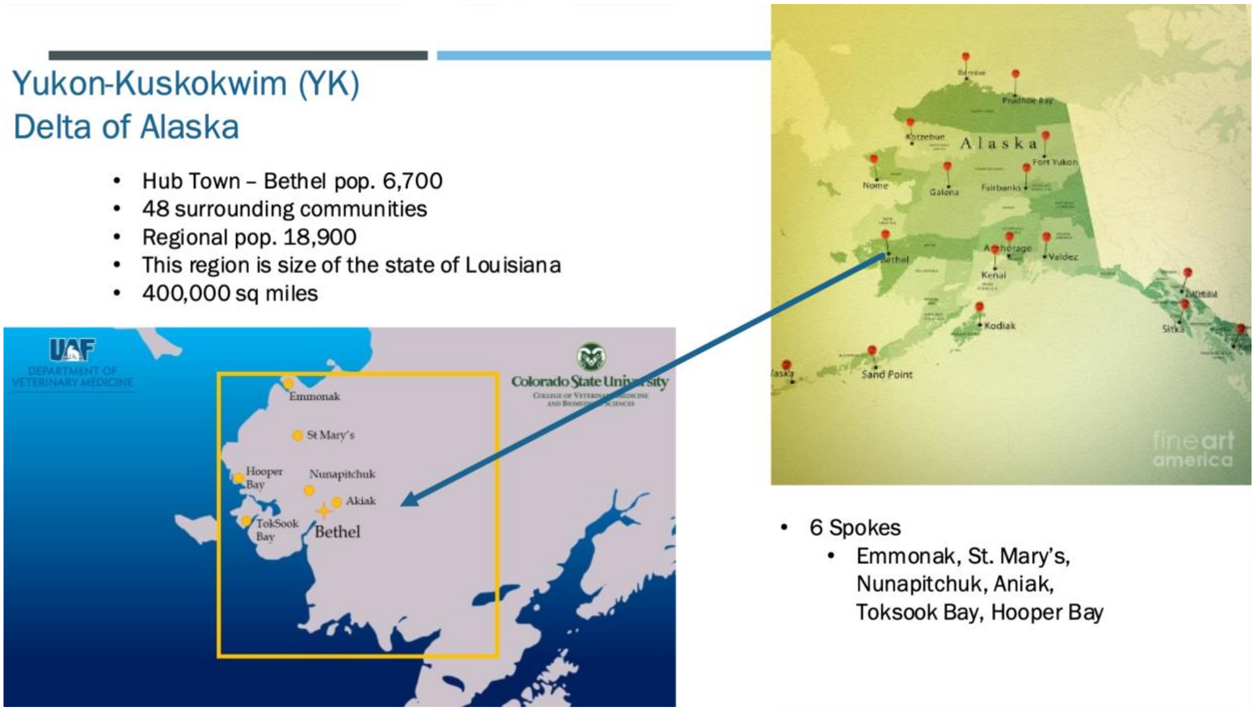

Hub Outpost Project

A summary and history of the project

In 2017, Drs. Arleigh Reynolds of the University of Alaska-Fairbanks (UAF) and Danielle Frey of Colorado State University (CSU) created a program addressing One Health in rural Alaska. From Bethel, Alaska, the program has partnerships with the surrounding villages in the Yukon-Kuskokwim (YK) Delta and capitalizes on Dr. Reynolds' regional expertise with dogs in Alaska and Dr. Frey's knowledge of working with communities experiencing barriers to veterinary care. Upon receiving funding from founding partner PetSmart Charities (PSC), the program officially commenced, and Dr. Laurie Meythaler-Mullins became the Community Outreach and Public Health Veterinarian (COPHV) responsible for coordinating the project and providing the veterinary services for the program in the YK Delta.

Dedicated to providing preventive veterinary care for companion animals in the YK Delta of Alaska, the HOP team works with owners who may be experiencing financial and geographical barriers to veterinary care for their pets. Villages in the YK Delta are remote

and off the road system, creating additional challenges for veterinarians trying to access the communities and for the transportation of animals to Bethel for veterinary care. Through a Hub and Spoke model, the HOP program provides access to veterinary care for nearly 48 rural communities by traveling via small plane, boat, or snowmachine to reach isolated people and animals.

The HOP team provides judgment-free care and supports the health of communities through the health of the animals by controlling transmission of zoonotic disease through vaccination and parasite treatments, and population management through spay/neuter surgery. Additionally, this program engages veterinary students interested in accessible veterinary care and rural medicine. With a USDA Veterinary Services Grant Program (VSGP) Education, Extension and Training (EET) grant, the HOP program expands the current local community school education programming to include curricula designed to share knowledge about One Health and medical professions with high school students. All community school projects are being designed to be culturally sensitive and inclusive, while working with local community members to include materials and concepts inclusive of the Yu'pik language and local spiritual and cultural beliefs.

Programs within the Project

- Preventive Health Care Veterinary Services
    - In Bethel
    - Village Community Visits
- Educational programs:
    - DVM student externships
    - One Health Veterinary Internship
    - School visits for all grades
    - High School focused education program
    - Public Facing Website
- Information Gathering
    - Survey

Bethel, the transportation and healthcare hub of the region, is a town of approximately 6,700 people with a strong Alaskan Native Yup'ik culture. It is surrounded by water and tundra, and home to over 240 bird species. In the summer there is strong salmon run and everyone is fishing. In the fall people are berry picking and in winter dog sled racing.

From Bethel, we will travel in a bush plane or boat to different villages, and provide basic public health veterinary services. Our veterinary team will be Dr. Laurie (lead veterinarian) and veterinary student externs.

In Bethel

Please know your cell phone will not work once you arrive in Bethel. You will have access to a phone to let loved ones know you arrived safely, but prior to travel please let them know your

communication will be sporadic. You may have access to internet in Bethel, in the village there will be no internet access.

Transportation in Bethel is actually very accessible; there is a vibrant taxi service- yet be certain to bring cash, as one way rides are around ~$15 per person. There may be times you need to taxi. The taxi service we use is Kusko Cab, 907-543-2169. We will try and carpool whenever possible.

There are no chain restaurants in Bethel. There's decent pizza, burger, and Asian restaurants, yet one pizza can cost around $40, and a one sushi roll around $30. There are two grocery stores here, and you can generally find things there you need, but expect to pay 2-3 times as much as you would in the Lower 48. For example, a gallon of milk here is around $13.

Students have found it helpful to pack some food, snacks, and instant coffee. You can pack them in your checked luggage. While we are in Bethel you will be responsible for your own food/meals; when we travel to a village the program will provide your food. *We plan and pack all of our village food.* IF THERE IS SOMETHING YOU CANNOT EAT- Dr. Laurie needs to be notified ASAP.

There is one hotel and a few bed and breakfasts in Bethel, but with prices around $250/night, we will work on finding other low to no-cost lodging options. You will likely be staying with a host family. If you do stay with a host family, please be mindful that living in Bethel is very expensive. Internet is pay-per-use, and limited water has to be delivered to the house- so download your shows to stream before arriving in Bethel, and don't take too long of a shower ☺ Please also look for ways you can help around the house!

Working in the region
The Yukon-Kuskowkim Delta is home to an Alaska Native population whom speak their native language of Yup'ik. English is also fluently spoken.

Your experience will seem much more like working in a developing country than a part of the United States. Some past students have compared their experience to Rural Area Veterinary Services (RAVS) trips. Other students have commented on this rotation feels like a "veterinary camping trip"; we sleep in sleeping bags, might not have running water, pack in and out all our own food, and for the most part are cut off from phone and internet service.

The state of human and animal lives with whom we work are very different from those you may have previously experienced. I do demand my externs keep an open mind and heart during your time here, and responsibly and respectfully represent veterinary medicine.

Since food is so expensive to ship in, a large part of the culture here is a traditional subsistence lifestyle; fishing, hunting and gathering. My favorite foods have been salmon, moose meat, and salmon berries.

Please note- Bethel is a “damp” community, meaning alcohol can be brought in, yet cannot be purchased in town. All other villages are “dry”- it is illegal to bring in or consume alcohol in these communities.

We have a partnership with the human health care provider of the region, the Yukon-Kuskokwim Health Care Corporation (YKHC). YHKC provides human health care to this region and its 48 villages.

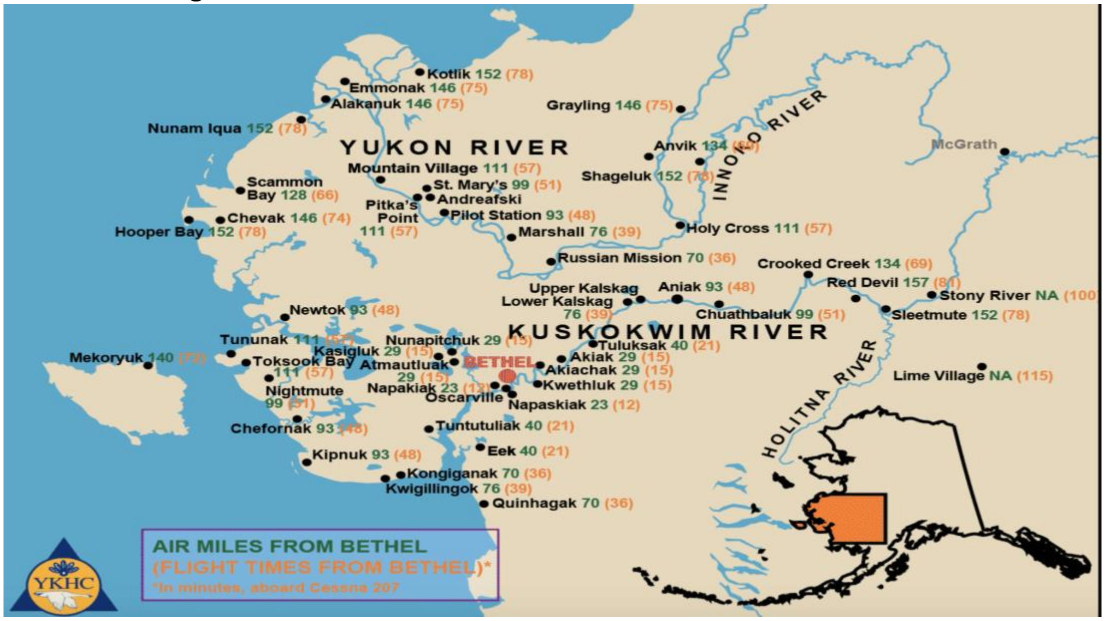

There is a veterinarian, Dr. Jessica Klejka, who travels into Bethel and opens her general practice four days a month. Knik Animal Clinic in Bethel does a wonderful job in Bethel, and we do not want our work to interfere with her business. The limited veterinary work we complete in Bethel is for income-qualifying families.

Our outreach clinics are focused on villages that may have not have ever seen a veterinarian, or if so quite a bit of time has passed. They are geographically isolated, and for most families it is not financially possible to travel via plane to Bethel or Anchorage to access veterinary care.

The program will book and pay for your village travel. Strict weight restrictions on small planes and the boat limit what we can take to a village with us. Duffel bags (especially a water-proof duffel) are ideal for your gear. More on what to pack will follow. Be aware, when you are checking in for a village flight, we will personally be weighed or publicly asked to declare our body weights.

In a village, we stay for no-cost at the YKHC Clinic. There is typically have a room with bunk beds (bring your own sleeping bag), a dorm fridge and microwave. Expect close quarters. There is running water and a shower- although your shower may be cold. Ask before you drink the water from the sink. We travel with water filters, and in some communities we need to use these.

We will do surgery indoors- BINGO Halls and Community Centers commonly. The surgeries are performed with injectable anesthesia and minimal equipment; yet we do maintain standards of care that are possible when completing this type of field work. At the start of your externship Dr. Laurie will ask about your previous surgical experience and what sort of surgical experience you hope to have while here, as well as other goals you may have.

In a village, we will walk house to house to give vaccinations. Be prepared to get dirty and wet. It will rain or snow, and mud boots are essential spring through fall. If you do not know what XTRATUF Boots are, Google them! Alaskan's live in those spring through fall. Check the temperature before you pack- in the summer a rain jacket and jeans work. In the winter you will need snow boots, a parka, gloves, face mask/hat. No matter which season, YOU WILL BE COLDER THAN YOU THINK, so when you pack, think warm and wind-proof. Nothing nice is necessary!

For surgery, scrubs are appropriate. I bring a separate pair of comfortable shoes (Crocs) to wear during surgery.

You will have the opportunity to complete spay and neuters, so review surgical techniques prior to arrival. Please note 99% of our surgeries are on dogs. Some of the more complicated spays Dr. Laurie will complete. We will work long days. We are aware that you are learning, and are here to support you. We hope to push you in the "growth" zone, but avoid pushing you to the "panic" zone!

Here is a VERY TENTATIVE general itinerary. This will change. Plans in rural Alaska can change hourly. Please know that flights will get cancelled. We may get weathered in a village for three days. Our gear might not make it. Electricity during surgeries goes out (pack head lamps!). Once in a village, we will not have internet access, and phone access may be spotty. Bring humor, patience, flexibility, and a good book, card game, downloaded movies, etc.

i. Typical CSU Rotation Schedule
   1. Day 1- students arrive in Bethel. Pick up from the airport and transport them to the host family with whom they are staying.
   2. Day 2- pick the students up from the host family and head out for village travel. When we arrive in the village we need to secure keys- keys from YKHC for lodging, and from the tribal council and the building we will be hosting our clinic. We will ask our arrival be announced over VHF radio and Facebook. We also need to secure transportation if lodging and working spaces are far apart. We will unpack, then if weather and daylight allows, we will start door-to-door vaccinations.
   3. Day 3- village school visit and door-to-door vaccination.
   4. Day 4- village surgery day
   5. Day 5- village surgery day
   6. Day 6- village surgery day

7. Day 7- return to Bethel. When we arrive in Bethel we will head to our working space in Bethel (the dog pound) to unpack and autoclave instruments. We will drive the students to the grocery store to buy food for their remaining time in Bethel. Note: this day may used as a buffer day if we are weathered in a village.
8. Day 8- pick up students, surgery day in Bethel for income-qualifying community members. Note: this day may used as a buffer day if we are weathered in a village.
9. Day 9- pick up students, surgery day in Bethel for income-qualifying community members. Note: this day may used as a buffer day if we are weathered in a village. Rotation debriefs, reviewing grading and turn in assignments. Transport students to airport.
10. Day 10- students depart Bethel

Expectation of veterinary externs

Veterinary externs will be expected to actively participate the all the HOP activities during their rotation. This includes, yet is not limited to:

1. Community outreach and education about veterinary medicine
2. Community education and administration of vaccinations
3. Community education and administration of anti-parasitics
4. Community education about spay/neuter surgery
5. Completion of spay and neuter surgeries
6. Set up and take down of MASH-style veterinary clinic
7. Surgical prep and medication administration
8. Recovery
9. Anesthesia
10. Completion of the Reflection Assignment

Reflection Assignment: Please pick a significant photo taken during your rotation and write the story behind why it is meaningful to you. Email this to Dr. Laurie at the completion of your rotation

Village Packing List

Boots- mud boots in summer, winter boot other seasons
Rain jacket in summer, heavy winter coat/parka all other seasons
Rain pants in summer, snow pants all other seasons
Face mask/balaclava in winter
Sunglasses (bring in winter as well for snow machine travel)
Warm hat
Warm gloves (wool is best- keeps your fingers warm even when wet)
Warm socks (wool is best)
Scrubs for surgery
Comfortable shoes for surgery
Surgical hat
Sleeping bag

Head Lamp
Entertainment for down time- downloaded movies, books, etc.
Snacks
Instant coffee/tea
Coffee/tea travel mug
Nalgene/water bottle
Towel
PJ’s
Extra change of clothes
Toiletries
You may wish to bring pack extra gear for Bethel or travel after your rotation- that is fine! This can be left in Bethel while we are in a village.

This is an awesome, sometimes challenging, and always worthwhile experience. We are very excited to work alongside you. Please don’t hesitate to reach out with questions.
