# Supplementary material for: Beyond clinical skills: student-reported impacts of a veterinary public health externship in rural Alaska
Source: Front Vet Sci. 2025 Dec 19;12:1613867. doi: 10.3389/fvets.2025.1613867 (PMC12758408; doi:10.3389/fvets.2025.1613867)
Supplement: Supplementary file 3 [file Data_Sheet_3.pdf]

CSU Alaska Hub Outpost Project Participant Survey

Dear participant,
We invite you to participate in our survey about the Off-Site Learning Opportunities (OSLO), programs offered by Dr. Danielle Frey in the Colorado State University (CSU) Doctor of Veterinary Medicine (DVM) program. We would like to request your feedback through this survey about your initial interest in and experiences of the opportunity. While there are no direct benefits to you, this will help to improve and evaluate the program. The survey may take about 10-20 minutes depending on the level of detail you provide.

Your participation in the study is voluntary. If you decide you do not want to participate in the study, you may withdraw your consent and stop participation at any time.

If you have any questions, please contact Allyce Lobdell at Allyce.Lobdell@colostate.edu or Dr. Danielle Frey at Danielle.Frey@colostate.edu.

Sincerely,
Dr. Danielle Frey
Dr. Laurie Meythaler-Mullins
Allyce Lobdell

○ I consent to participate and would like to continue

○ I do not consent and would not like to continue

Please enter your name (First Last), or choose to complete the survey anonymously

○ My name is ____

○ I prefer to answer anonymously

Did you participate in a CSU Off Site Learning Opportunity (OSLO) as a CSU veterinary student or as a licensed veterinary professional participant? These are programs offered by Dr. Danielle Frey in the Doctor of Veterinary Medicine program.

○ Yes

○ No

In which capacity did you participate? If you participated in more than one way, please select the role for which you received the survey request. You are also welcome to complete this survey again to share your experiences in other roles.

- ◯ CSU Veterinary Student
- ◯ Licensed Veterinary Professional

Please select the experience in which you participated. If you participated in more than one, please select the experience for which you received the request for you to complete the survey. You can return to complete the survey for additional experiences.

- ◯ Community Outreach in the YK Delta, Alaska
- ◯ One Health Conservation on the Pribilof Islands, Alaska

Select the year you participated. If you're unsure, make your best guess.

- ◯ 2019
- ◯ 2020
- ◯ 2021
- ◯ 2022
- ◯ 2023
- ◯ 2024

[Next 3 questions are Professional Participant Questions only]
What was appealing about this opportunity when you agreed to participate?

____

____

____

____

____

What did you expect gain?

What was the primary reason you participated?

[Next 3 questions are Student Questions only]
What was appealing about this externship when you applied?

What did you expect to learn from the experience when you applied?

What was the primary reason you participated?

____

____

____

____

____

Next we would like to ask you some questions about your experiences during the Off-Site Learning Opportunity.

Which of your veterinary clinical skills were significantly impacted through participation in this opportunity? Please mark any that apply.

- ☐ Client communication
- ☐ Collegial communication
- ☐ Teamwork
- ☐ Surgical skills
- ☐ Patient handling
- ☐ Vaccination administration
- ☐ None of the above
- ☐ Other (you will be asked more about this in a moment)

[Triggered questions if category was selected in question above]
If you're able, please describe how do you think participation in this opportunity impacted your **client communication** skills?

____

____

If you're able, please describe how do you think participation in this opportunity impacted your **collegial communication** skills?

If you're able, please describe how do you think participation in this opportunity impacted your **teamwork** skills?

If you're able, please describe how do you think participation in this opportunity impacted your **surgical skills**?

If you're able, please describe how do you think participation in this opportunity impacted your **patient handling** skills?

If you're able, please describe how do you think participation in this opportunity impacted your **vaccination administration** skills?

If you're able, please describe how do you think participation in this opportunity impacted any **other clinical skills** you may have gained?

Now we would like to ask you about impacts to your knowledge from participating in this experience.

Was your perception or knowledge of any of the topics below impacted through participating in this externship? Please mark any that apply.

- ☐ Working in rural communities
- ☐ Working with limited resources
- ☐ Zoonotic diseases
- ☐ Public Health
- ☐ Epidemiology
- ☐ Food insecurity
- ☐ Food safety
- ☐ Resource restricted, geographically isolated, and nontraditional clinic spaces
- ☐ None of the above
- ☐ Other (you will be asked more about this in a moment)

[Triggered questions if category was selected in question above]
If you're able, please describe how participation impacted your perception or knowledge of **working in rural communities**?

____

____

____

____

____

If you're able, please describe how participation impacted your perception or knowledge of **working with limited resources**?

If you're able, please describe how participation impacted your perception or knowledge of **zoonotic diseases**?

If you're able, please describe how participation impacted your perception or knowledge of **public health**?

If you're able, please describe how participation impacted your perception or knowledge of **epidemiology**?

If you're able, please describe how participation impacted your perception or knowledge of **food insecurity**?

If you're able, please describe how participation impacted your perception or knowledge of **food safety**?

If you're able, please describe how participation impacted your perception or knowledge of **resource restricted, geographically isolated, and nontraditional clinic spaces**?

____

____

____

____

____

You selected that there was another topic in which your perception or knowledge was impacted through participating in this experience. If you're able, please describe what that is/those are here.

____

____

____

____

____

Finally, the next set of questions asks you to reflect and share about whether this experience has had any long term impacts on you. ***If you have participated recently and the question does not yet apply, please write "NA."**

Do you believe this experience has had a lasting impact on you since participating?

- ○ Yes
- ○ No

If you're able, please describe how this experience has had a lasting impact.

____

____

____

Has this experience impacted your ability to work with clients who differ from yourself (for example, in terms of cultural background, socioeconomic status, etc.)?

- ○ Yes
- ○ No
- ○ Unsure

You indicated this experience has or may have impacted your ability to work with clients who differ from yourself. If you're able, please describe how this experience made an impact in that way?

Do believe you apply the One Health concept to how you practice veterinary medicine?

- ○ Yes
- ○ Unsure
- ○ No

You indicated that you do or are unsure if you do apply the One Health concept to how you practice veterinary medicine.

If you're able, please describe how, or if, this experience had an impact on how you apply, try to apply, or think about applying the One Health concept in how you practice veterinary medicine?

____

____

____

____

____

Did this experience provide you with any of the following tools to work in **rural regions**, specifically?

- ☐ Confidence
- ☐ Surgical Skills
- ☐ Ability to practice in low-resourced situations
- ☐ Ability to work in geographically isolated spaces
- ☐ Ability to work in nontraditional clinic spaces
- ☐ Communication
- ☐ Professional collaboration
- ☐ Client connection and rapport
- ☐ Other ________________________________
- ☐ None of the above

Did you learn anything about yourself, your profession, or your professional/personal aspirations while engaged in this experience? If so, please share below.

____

____

____

____

____

Would you like to share any other ways that this experience impacted you or your approach to veterinary medicine? If so, please describe below.

____

____

____

____

____

We would like to ask you some demographic and other questions about yourself. Please answer as you feel comfortable. Having this information helps us to improve our programming based on the demographics of our participants. We appreciate any information you share.

During or after which year of the veterinary curriculum did you participate in the experience? For some examples, you may have participated during your 4th year (select 4th), during your 1st year at UAF (select 1st), or the summer after your 2nd year (select 2nd).

○ 1st (first) year

○ 2nd (second) year

○ 3rd (third) year

○ 4th (fourth) year

Were/are you enrolled in the UAF 2+2 program during your time in the CSU DVM program?

- ◯ Yes
- ◯ No

Were you attending UAF during your externship?

- ◯ Yes
- ◯ No

Have you graduated since participating?

- ◯ Yes
- ◯ No

In what year did you graduate from veterinary school?

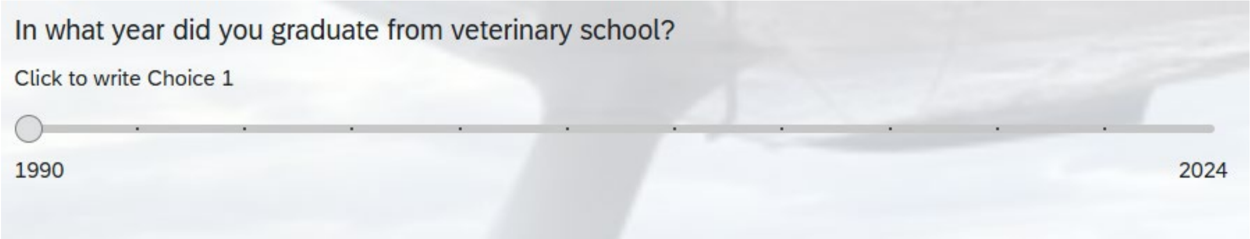

Are you a practicing veterinarian?

- ◯ Yes
- ◯ No
- ◯ Partially. Please explain. ____

Please select the answer that BEST describes your veterinary practice:

- ○ Small animal general practice
- ○ Small animal specialty or referral practice
- ○ Small animal emergency practice
- ○ Shelter/rescue
- ○ Mixed practice, small animal focused
- ○ Mixed practice, large animal focused
- ○ Livestock
- ○ Equine
- ○ Exotics
- ○ Other: (please specify) ____

What type of work are you doing now?

- ☐ Veterinary-related teaching
- ☐ Veterinary-related administration
- ☐ Veterinary-related research
- ☐ Non-veterinary related work. Please describe.

____

What was your travel experience at the time of participating in the OSLO (Off Site Learning Opportunity)?

	Never	Limitedly (no more than once/year)	Moderately (1-2 times per year)	Extensively (more than 2 times per year)
I traveled outside of my home region **before** this experience	○	○	○	○
I experienced domestic travel **before** this experience	○	○	○	○
I experienced international travel **before** this experience	○	○	○	○

What has your travel experience been like since participating in the OSLO?

	Never	Limitedly (no more than once/year)	Moderately (1-2 times/year)	Extensively (more than 2 times/year)
On average, I have traveled outside of my home region **since** this experience	○	○	○	○
On average, I have experienced domestic travel **since** this experience	○	○	○	○
On average, I have experienced international travel **since** this experience	○	○	○	○

What was your age at the time of this experience? If you're unsure, make your best guess. (Age updates on the right of slider.)

Click to write Choice 1

18 70

What is your age now? (Age updates on the right of slider.)

Click to write Choice 1

18 70

Choose one or more race/ethnicities that you consider yourself to be:

- ☐ White
- ☐ Black or African American
- ☐ Native American/American Indian or Alaska Native
- ☐ Hispanic or Latino/e
- ☐ Asian/Asian American
- ☐ Native Hawaiian or Pacific Islander
- ☐ Other ____________________
- ☐ Prefer not to say

Which of the below best describes your current gender identity?

- ○ Man
- ○ Woman
- ○ Nonbinary
- ○ Genderqueer or genderfluid
- ○ Transgender woman
- ○ Transgender man
- ○ A gender not included here

____

In what city and state do you currently reside?

____

What social class do you consider being brought up within? See link for quick run down of social classes.

- ◯ Lower class
- ◯ Working class
- ◯ Middle class
- ◯ Upper class

What social class do you consider being in now? See link for quick run down of social classes.

- ◯ Lower class
- ◯ Working class
- ◯ Middle class
- ◯ Upper class
